# Erythroderma: clinical and etiological study of 88 cases seen in a tertiary hospital over 25 years^☆^

**DOI:** 10.1016/j.abd.2023.07.014

**Published:** 2024-04-23

**Authors:** Rogério Nabor Kondo, Betina Samesima e Singh, Milene Cripa Pizatto de Araújo, Victória Prudêncio Ferreira, Jessica Almeida Marani, Airton dos Santos Gon

**Affiliations:** Department of Internal Medicine, Universidade Estadual de Londrina, Londrina, PR, Brazil

Dear Editor,

Exfoliative erythroderma (EE), exfoliative dermatitis, or simply erythroderma, first described by Von Hebra in 1868, is a rare disorder in which erythema and desquamation occur, involving more than 90% of the body surface.^1^

Previous studies have shown the main etiology to be pre-existing (or underlying) dermatoses, followed by medications and, less commonly, neoplasms.^2^, ^3^, ^4^ We assume that increased use and access to new drugs, and drug interactions, especially in the elderly, may be modifying the epidemiology, with drugs being the main etiology of EE.

To investigate this hypothesis, an observational and retrospective study was carried out by reviewing the medical records of patients with EE diagnosed at a university hospital of Universidade Estadual de Londrina, from February 1, 1996 to February 1, 2021.

Data were collected in forms developed by the researchers themselves. The collected information was compiled in an Excel spreadsheet for statistical analysis. The Stata® program (version 13.0, Statacorp Texas) and Jamovi 1.6.15 were used for the statistical analysis. Statistical significance values (p-value) < 0.05 and a 95% confidence interval were considered.

Table 1 shows the main findings of the study. There were a total of 88 individuals, 52 males (59.09%) and 36 females (40.91%), a ratio of 1.4:1, p = 0.06. The mean age of the individuals was 44.72 years (range: 0‒84 years). The majority of the individuals were white (n = 74 or 84.09% [95% CI 76.50%–94.39%]) versus non-white (n = 14 or 16.09% [95% CI 13 .71%‒66.68%]) with p = 0.03, data in accordance with the literature.^2^, ^3^

**Table 1:** Demographic and clinical characteristics of erythroderma patients.

Characteristics	n = 88
Age, years	
Mean ± SD	44.72 ± 25.08
Minimum – Maximum	0 – 84
Gender, n (%)	
Male	52 (59.09)
Female	36 (40.91)
Ethnicity, n (%)	
White	74 (84.09)
Brown	7 (7.95)
Black	3 (3.41)
Yellow	4 (4.55)
Etiologies, n (%)	
Dermatoses	43 (48.86)
Drug reactions	41 (46.60)
Cutaneous lymphoma	2 (2.27)
Undetermined	2 (2.27)
Main dermatoses, n (%)^a^	
Atopic dermatitis	14 (15.91)
Psoriasis	12 (13.63)
Contact dermatitis	9 (10.23)
Pityriasis rubra pilaris	3 (3.41)
Others^b^	5 (5.68)
Main classes of drugs, n (%)^c^	
Antimicrobials	13 (14.77)
Anticonvulsants	12 (13.63)
Laboratory tests, n (%)	
Altered^d^	64 (72.73)
Deaths, n (%)^e^	
Yes	4 (4.55)

a Percentage in relation to n = 88.

b Others: Non-bullous congenital ichthyosiform erythroderma (2/43); Netherton syndrome (1/43); Seborrheic dermatitis (1/43); Darier's disease (1/43); Staphylococcal scalded skin syndrome.

c Percentage in relation to n = 88.

d 72.73% of patients had at least one laboratory abnormality.

e Deaths due to cutaneous lymphoma (2/88), drug reaction classified as drug reaction with eosinophilia and systemic symptoms/DRESS (2/87).

As for the etiology (Table 2), pre-existing dermatoses (n = 43 [48.86%]) followed by drug reactions (n = 41 [46.60%]) were the main causes of EE, with no significant difference amongst them (p = 0.88), but there was a significant difference when they were added together (n = 84 [96.55%]) and compared to other etiologies (n = 4 [4.55%]) and p < 0.001. Previous studies point to the group of pre-existing dermatoses as the main cause but with a greater difference in relation to drug reactions.^2^, ^3^, ^4^, ^5^, ^6^

**Table 2:** Erythroderma according to etiology (n = 88).

Etiology	Number	%
**I) Dermatoses**		
Atopic dermatitis	14	15.90
Psoriasis	12	13.63
Contact dermatitis	7	7.95
Pityriasis rubra pilaris	3	3.41
Non-bullous congenital ichthyosiform erythroderma	2	2.27
Seborrheic dermatitis	1	1.14
Infective dermatitis	1	1.14
Circumflex linear ichthyosis	1	1.14
Follicular keratosis	1	1.14
Staphylococcal scalded skin syndrome	1	1.14
**II) Drug reactions**		
Carbamazepine	7	7.94
Ceftriaxone	3	3.41
Phenobarbital	3	3.41
Valproic acid	2	2.27
Amoxicillin	2	2.27
Sulfamethoxazole + trimethoprim	2	2.27
Allopurinol	2	2.27
Sulfasalazine	2	2.27
Acetylsalicylic acid	1	1.14
Azithromycin	1	1.14
Codeine	1	1.14
Contrast	1	1.14
Dipyrone	1	1.14
Levofloxacin	1	1.14
N-acetylcysteine	1	1.14
Piroxicam	1	1.14
Norfloxacin	1	1.14
Promethazine	1	1.14
Vancomycin	1	1.14
Undefined^a^	5	5.68
**III) Neoplastic**		
Cutaneous lymphoma^b^	2	2.27
**IV) Undetermined**	2	2.27
**Total**	88	100

a Drug-related cause, but the patient did not remember the medication or was using a combination of several medications, such as in the treatment of *H. pylori*.

b Case of cutaneous T-cell lymphoma and another of Sézary syndrome.

Atopic dermatitis (AD) was more prevalent than psoriasis (Pso; n = 14 [15.90%] vs. n = 12 [13.63%], p = 0.81), which is not in accordance with the literature, which shows the opposite.^6^ One hypothesis would be the influence of access to immunobiological therapies for patients with Pso through the Unified Health System (SUS, Sistema Único de Saúde) in Brazil, which allows the early treatment of severe cases, preventing progression to the erythrodermic form.^7^ On the other hand, there is an assumption that the lack of more effective and accessible medications through SUS for severe AD conditions may be causing EE in these treatment-refractory patients.^8^

In agreement with the literature,^1^ antibiotics and anticonvulsants were the main causes (Table 1, Table 2). Carbamazepine, although not statistically significant when compared to all medications, was the most frequent drug involved (n = 7 [7.94%] vs. n = 34 [38.64%], p = 0.24).

Anticonvulsants are the main causes of “drug reaction with eosinophilia and systemic symptoms” (DRESS), which is induced by medications and presents as an extensive rash-like eruption, associated with lymphadenopathy, hepatitis, hematological abnormalities with eosinophilia and atypical lymphocytes, and may involve other organs such as lung, heart, and kidneys.^9^ The condition can have a bad outcome, including death, as happened in two cases of the present study.

The mean age of the dermatosis group was younger than that of the drug reaction group (n = 43; mean = 38.3 years (95% CI 30.50–46.02)] versus (n = 41; mean = 48. 7 years (95% CI 41.20–56.11], p < 0.001; Fig. 1) we hypothesize that it is due to the early onset of diseases such as AD and childhood seborrheic dermatitis or presentation at birth, such as non-bullous ichthyosiform erythroderma. As for drug reactions, on the contrary, with advancing age, there is a greater need and frequency of drug use, and chances of drug interactions and reactions.

**Figure 1:**
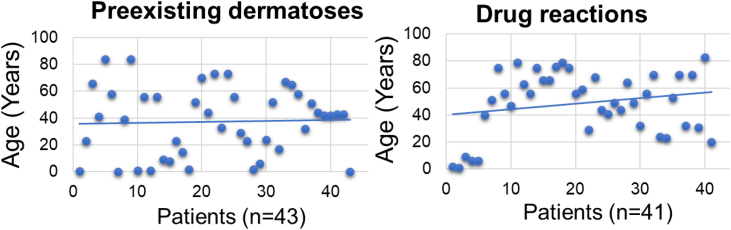
Scatter plot depicting ages in the dermatosis and drug reactions groups.

Previous studies have shown variable ratios between the frequency of underlying dermatoses and drug reactions, of 2.6:1 to 6.4:1, as causes of EE. In comparison to previous studies, the highest values were observed in a study from Singapore (Table 3). The present findings seem to indicate a tendency for drug reactions to become increasingly more frequent and consequently one of the main causes of EE. On the other hand, effective and accessible therapies for pre-existing dermatoses can potentially reduce hospitalizations due to EE.

**Table 3:** Association between cases of dermatoses and drug reactions.

Study (year)	N	Dermatoses	Drug reactions	Proportion
Akhyani et al. (2005)	97	59.7	21.6	2.7:1
Fernandes et al. (2008)	170	58.2	21.7	2.6:1
Tan et al. (2014)	30	68.9	10.7	6.4:1
Cesar et al. (2016)	103	65.0	18.4	3.5:1
Miyashiro et al. (2020)	309	46.2	12.3	3.7:1
Present study	88	48.9	46.6	1.0:1

N represents the number of patients; proportion represents the association between dermatoses and drug reactions.

Elderly people have a higher frequency of diseases, and use more medications which may interact. Additionally, in senescence there may be changes in pharmacokinetics, affecting drug metabolism and clearance, which increase the chances of developing drug reactions.^10^

In the literature, cases with undetermined etiology vary between 3.9% and 16.8%.^2^, ^3^, ^4^, ^5^, ^6^ The present study showed a low prevalence (2.27%), one of the reasons being the exclusion of eight undetermined etiology cases after reviewing the medical records. Of these, seven patients had presented EE before the starting date of the study but continued the follow-up. Another undetermined case was excluded because less than 90% of the body surface was affected.

Neoplasms (n = 2 [2.27%]) and paraneoplastic syndromes (n = 0 [0%]) had a low frequency in the present sample, but this is in accordance with the literature that describes it as ranging from zero to 17.8%.^6^ Mycosis fungoides (MF) and Sézary syndrome (SS) were the neoplastic causes.

Data on the prevalence of mortality in EE are still very scarce,^6^ but the present sample showed a prevalence of 4.55%, with two cases of lymphoma (MF and another with SS) and two patients with DRESS.

The limitations of the present study include selection bias and sample size.

Controlling underlying dermatoses with more effective and accessible therapies can substantially lead to a reduction in EE caused by this etiology. On the other hand, population aging associated with increased frequency of use and access to new medications may maintain or increase drug reactions as the main cause of EE.

## Financial support

None declared.

## Authors’ contributions

Rogério Nabor Kondo: Design and planning of the study; data collection, or analysis and interpretation of data; statistical analysis; drafting and editing of the manuscript or critical review of important intellectual content; collection, analysis and interpretation of data; effective participation in research orientation; critical review of the literature; approval of the final version of the manuscript.

Betina Samesima and Singh: Data collection, or analysis and interpretation of data; drafting and editing of the manuscript or critical review of important intellectual content; collection, analysis and interpretation of data; critical review of the literature; approval of the final version of the manuscript.

Milene Cripa Pizatto de Araújo: Data collection, or analysis and interpretation of data; drafting and editing of the manuscript or critical review of important intellectual content; collection, analysis and interpretation of data; critical review of the literature; approval of the final version of the manuscript.

Victória Prudêncio Ferreira: Data collection, or analysis and interpretation of data; drafting and editing of the manuscript or critical review of important intellectual content; collection, analysis and interpretation of data; critical review of the literature; approval of the final version of the manuscript.

Jessica Almeida Marani: Data collection, or analysis and interpretation of data; drafting and editing of the manuscript or critical review of important intellectual content; collection, analysis and interpretation of data; critical review of the literature; approval of the final version of the manuscript.

Airton dos Santos Gon: Design and planning of the study; statistical analysis; drafting and editing of the manuscript or critical review of important intellectual content; collection, analysis and interpretation of data; intellectual participation in the propaedeutic and/or therapeutic conduct of the studied cases; critical review of the literature; approval of the final version of the manuscript.

## Conflicts of interest

None declared.

## References

[1] Tso S., Satchwell F., Moiz H., Hari T., Dhariwal S., Barlow R. (2021). Erythroderma (exfoliative dermatitis). Part 1: underlying causes, clinical presentation and pathogenesis. Clin Exp Dermatol.

[2] Akhyani M., Ghodsi Z.S., Toosi S., Dabbaghian H. (2005). Erythroderma: a clinical study of 97 cases. BMC Dermatol.

[3] Fernandes N.C., Pereira F.S.M., Maceira J.P., Cuzzi T., Dresch T.F.L.R., Araújo P.P. (2008). Eritrodermia: estudo clínico-laboratorial e histopatológico de 170 casos. An Bras Dermatol.

[4] Tan G.F., Kong Y.L., Tan A.S., Tey H.L. (2014). Causes and features of erythroderma. Ann Acad Med Singap.

[5] Cesar A., Cruz M., Mota A., Azevedo F. (2016). Erythroderma. A clinical and etiological study of 103 patients. J Dermatol Case Rep.

[6] Miyashiro D., Sanches J.A. (2020). Erythroderma: a prospective study of 309 patients followed for 12 years in a tertiary center. Sci Rep.

[7] Carrasquillo O.Y., Pabón-Cartagena G., Falto-Aizpurua L.A., Santiago-Vázquez M., Cancel-Artau K.J., Arias-Berrios G. (2020). Treatment of erythrodermic psoriasis with biologics: a systematic review. J Am Acad Dermatol.

[8] Pereyra‐Rodriguez J.J., Dominguez‐Cruz J., Armario‐Hita J.C., Villaverde R.R. (2021). 104‐week safety and effectiveness of dupilumab in the treatment of severe atopic dermatitis. The experience of 5 reference dermatology units in Spain. An Bras Dermatol.

[9] Hama N., Abe R., Gibson A., Phillips E.J. (2022). Drug-induced hypersensitivity syndrome (DIHS)/drug reaction with eosinophilia and systemic symptoms (DRESS): clinical features and pathogenesis. J Allergy Clin Immunol Pract.

[10] Zazzara M.B., Palmer K., Vetrano D.L., Carfì A., Onder G. (2021). Adverse drug reactions in older adults: a narrative review of the literature. Eur Geriatr Med.

